# *Aspergillus terreus* obtained from mangrove exhibits antagonistic activities against *Pythium aphanidermatum*-induced damping-off of cucumber

**DOI:** 10.7717/peerj.7884

**Published:** 2019-10-21

**Authors:** Hanaa Al-Shibli, Sergey Dobretsov, Abdulrahman Al-Nabhani, Sajeewa S.N. Maharachchikumbura, Velazhahan Rethinasamy, Abdullah M. Al-Sadi

**Affiliations:** 1 Department of Crop Sciences, College of Agricultural and Marine Sciences, Sultan Qaboos University, Al-Khoudh, Muscat, Oman; 2 Department of Marine Science and Fisheries, College of Agricultural and Marine Sciences, Sultan Qaboos University, Al-Khoudh, Oman; 3 Center of Excellence in Marine Biotechnology, Sultan Qaboos University, Al-Khoudh, Oman; 4 Department of Pathology, College of Medicine and Health Sciences, Sultan Qaboos University, Al-Khoudh, Oman

**Keywords:** Biocontrol, Oomycete, Marine fungi, Mangrove, *Avicennia marina*

## Abstract

A study was conducted to investigate the potential of *Aspergillus terreus* obtained from *Avicennia marina* mangrove roots in inhibiting *Pythium aphanidermatum* and damping-off disease of cucumber. *Aspergillus terreus* exhibited in vitro inhibition of *Pythium aphanidermatum* growth. Electron microscope examination revealed that the antagonistic fungal isolate resulted in shrinking and groves in *Pythium* hypha. When *Aspergillus terreus* culture filtrate was added to *Pythium aphanidermatum*, it resulted in a significant increase (by 73%) in electrolyte leakage from *Pythium* hypha compared to the control, as well as significant reduction (by 71%) in oospore production. The *Aspergillus terreus* culture was also found to produce a cellulase enzyme, which is suggested to be involved in the antagonism against *Pythium aphanidermatum*. Adding *Aspergillus terreus* to soil infested with *Pythium aphanidermatum* significantly reduced percent mortality in cucumber seedlings by 70%. *Aspergillus terreus*, when applied alone on cucumber seedlings, did not show any suppressive effects on cucumber growth (length and fresh and dry weight). This appears to be the first report of isolation from mangrove of *Aspergillus terreus* with antagonistic activity against *Pythium aphanidermatum*-induced damping-off of cucumber. The study indicates that fungal isolates obtained from marine environments may serve as potential biocontrol agents against some plant pathogens.

## Introduction

*Pythium* species are a major cause of root rot and damping-off diseases in several vegetables, ornamentals, cereals, and other crops (Al-Sadi et al., 2011; Hatami et al., 2013; Ishiguro et al., 2014; Al-Mahmooli et al., 2015; Gossen et al., 2016). *Pythium aphanidermatum* is one of the most commonly distributed and aggressive species of *Pythium* in the world (Al-Mawaali et al., 2013; Ishiguro et al., 2014; Le, Smith & Aitken, 2016). It is the most common cause of damping-off and vine decline diseases of cucumber (Al-Sadi et al., 2011, 2012; Al-Mawaali et al., 2012, 2013). It has been reported to result in up to 50% mortality of cucumber seedlings and plants under favorable conditions (Stanghellini & Phillips, 1975; Al-Sadi et al., 2011).

Several methods have been developed for the management of *Pythium*-induced diseases. In arid and hot areas of the world, covering soil with transparent polyethylene sheets for a period of 2–8 weeks (solarization) has been effective in reducing populations of *Pythium* and some other fungal species in soil (Farraq & Fotouh, 2010; Kokalis-Burelle et al., 2017). Biofumigation has also shown to be effective, but it is less frequently used (Deadman, Al-Hasani & Al-Sa’di, 2006). Chemical control through the use of several fungicides such as mefenoxam and hymexazol is widely practiced (Al-Sadi, 2012; Matić et al., 2019). However, several studies have shown failure of chemical control due to several reasons, including the development of fungicide resistance, rapid biodegradation of fungicides in soil and delayed activation of the pathogen following application of the fungicide (Eckert, 1988; Al-Sa’di et al., 2008; Al-Sadi et al., 2012; Al-Balushi et al., 2018).

The use of biocontrol agents in managing soil-borne diseases is common in several areas of the world, especially under organic agriculture. Several biocontrol agents have been tested and used against *Pythium, Rhizoctonia*, and *Fusarium* species, including *Trichoderma harzianum* and *Pseudomonas aeruginosa* (Al-Hinai et al., 2010; Blaya et al., 2013; Halo, Al-Yahyai & Al-Sadi, 2018; Kazerooni, Rethinasamy & Al-Sadi, 2018). The biocontrol agents interfere with plant pathogens through the production of toxic metabolites and enzymes (e.g., cellulase), competition for food or space and/or direct parasitism (Bibi et al., 2017; Sharma, Salwan & Sharma, 2017; Vinale et al., 2017; Halo, Al-Yahyai & Al-Sadi, 2018; Zhang et al., 2018). Biocontrol agents are usually obtained from soils with heavy disease pressures, from plants grown in the same field or from wild plants (Lyu et al., 2015; Mayo et al., 2017; Raza et al., 2017). However, no previous attempts have been made to isolate a biocontrol agent against *Pythium aphanidermatum* from mangrove.

Marine fungi are considered critical and important component of the marine ecosystem (Tisthammer, Cobian & Amend, 2016). Marine fungi are found on a wide range of substrates, including sediments, algae, sand grains, submerge wood, animals, and plants (Overy et al., 2019).

Mangrove is a shrub/small tree that grows in saline environments. The mangrove *Avicennia marina* is present in coastal areas of several countries, including Oman, China, Iran, and others (Ghazanfar, 1999; Al-Nadabi & Sulaiman, 2018; Nasr et al., 2018; Tong et al., 2019). Mangrove has been reported to be rich in biological diversity (Dias et al., 2012). Previous studies showed that some fungal species are common in the rhizosphere and different parts of the mangrove tree (Sanka Loganathachetti, Poosakkannu & Muthuraman, 2017; Prihanto, Caisariyo & Pradarameswari, 2019). The saline environment, the special life cycle of marine fungi and the unique marine fungal-host interactions make them a valuable source of secondary metabolites (Balabanova et al., 2018; Deshmukh, Prakash & Ranjan, 2018).

This study was conducted to investigate whether fungal species associated with mangrove *Avicennia marina* could show antagonistic activities against *Pythium aphanidermatum*. Specific objectives were: (1) isolate and examine the efficacy of *Aspergillus terreus*, obtained from mangrove, on the inhibition of mycelial growth and morphology of *Pythium aphanidermatum*, (2) to its their efficacy in suppressing Pythium damping-off disease, and (3) to examine the production of cellulase enzyme by the fungal isolates. This study will lay the ground for future research on the utilization of marine fungi in managing crop diseases.

## Materials and Methods

### Sample collection

Breathing roots of mangroves were collected from three trees at Al-Qurum Area in Muscat Governorate, Oman. The collection of samples was done from three plants during October and November 2017. Three leaf/root/stem samples were collected from each plant. Samples (size ~5 cm) were washed with tap water and then cut into small pieces followed by surface sterilization with 1% sodium hypochlorite, and washing three times with sterile distilled water. The samples were then dried on sterilized filter papers. Next, four pieces (~1 cm^3^) from each sample were placed on Petri dishes containing 3% malt extract media (MEA) and 2.5% potato dextrose agar (PDA). The Petri dishes were incubated at room temperature (25 °C) for at least 10 days. Fungi were transferred to new plates. Pure fungal colonies were preserved on 2.5% PDA slants.

### Antagonistic activity of fungal isolates against *Pythium aphanidermatum*

The efficacy of fungal isolates obtained from mangrove roots in inhibiting mycelial growth of *Pythium aphanidermatum* (obtained and identified during a previous study, Al-Sadi et al., 2012) was investigated. A four mm diameter disc of 10 day-old culture of each fungal isolate was placed at the end of Petri dishes containing 2.5% PDA. A four mm disc of 3-day old *Pythium aphanidermatum* was placed on the other end of the Petri dish. Control plates had *Pythium aphanidermatum* discs on one end, without fungal isolates on the other end. Three replicates were used for the treatments and the control. The plates were incubated at 24 °C up to 10 days, and the experiment was repeated to confirm the results obtained. The size of the inhibition zone was measured after *Pythium aphanidermatum* reached the end of the plate in the control plate.

### Identification of the antagonistic *Aspergillus terreus* isolate

Since only one fungal isolate (*Aspergillus terreus*) from the previous experiment showed antagonistic activity against *Pythium aphanidermatum*, its identification was confirmed using sequences of the internal transcribed spacer region of the ribosomal RNA gene (ITS rRNA) (Al-Sadi, Al-Mazroui & Phillips, 2015). In order to do this, DNA extraction was done from 80 mg of freeze-dried fungal mycelium as described by Al-Sadi et al. (2012). Briefly, the fungal mycelium was ground with 30 mg sterile sand in an Eppendorf tube. Then 600 μl of lysis buffer was added and mixed with the sample. The sample was incubated at 65 °C for 30 min. After that, 600 µl of phenol chloroform isoamyl alcohol (25:24:1) was added followed by centrifugation at 10,000 rpm for 15 min. The last two steps were repeated twice. The supernatant was transferred into new 1.5 ml Eppendorf tubes and 300 µl of isopropanol and 10 µl of 3M NaOAc were added to the supernatant. The tube was inverted gently and then incubated at −20 °C for 20 min. After that, centrifugation was done for the samples at 10,000 rpm for 2 min by using a refrigerated micro centrifuge. The DNA was washed using 70% ethanol. Then, centrifugation was done for the mixture at 10,000 rpm for 2 min. Ethanol was discarded and the pellet was allowed to dry. The DNA was dissolved in 100 µl Milli-Q water and stored at −20 °C.

Amplification of the ITS rRNA region of the isolated fungi was done using the ITS4 (reverse: TCCTCCGCTTATTGATATGC) and ITS5 primers (forward: GGAAGTAAAAGT CGTAACAAGG) (White et al., 1990). The PCR reaction mixture and conditions were as described by Al-Sadi et al. (2012).

Sequencing was conducted at Macrogen Inc. (Seoul, Korea). Maximum likelihood analyses including 1,000 bootstrap replicates were run using RaxmlGUI v. 1.3 (Silvestro & Michalak, 2012). The *Aspergillus terreus* sequence was deposed at National Center for Biotechnology Information (NCBI) under the accession number MK680140.

### Effect of *Aspergillus terreus* isolate on *Pythium aphanidermatum* morphology

Further work was carried out on the *Aspergillus terreus* isolate by examining the effect of the fungus on *Pythium aphanidermatum* hyphal morphology, which was screened and examined using a Scanning Electron Microscope (SEM, JEOL, Japan JSE- 5600) (Goldstein et al., 2017). A piece of mycelium of about 0.5 cm at the inhibition zone was cut. Samples were put in fixative solution to fix the sample tissue. After 1 h of incubation samples were washed two times with buffer (Osmium Tetroxide), 10 min each time. Osmium Tetroxide was added again to the samples and incubated for 1 h to stain the sample lipids and then washed with distilled water. To remove extra water from the sample, ethanol was added to the samples in sequence at concentrations of 25%, 75%, 95% and absolute ethanol, 10 min for each concentration. An automatic drying machine was used to remove the remaining ethanol to reach critical drying point. Finally, the samples were coated with gold and then the samples were examined using the scanning electron microscope.

Differences in morphology between *Pythium aphanidermatum* hypha near the inhibition zone and the control *Pythium aphanidermatum* were examined. The focus was on hypha shape, cytoplasm content, and the end of hypha tips.

### Effect of *Aspergillus terreus* isolate on extracellular conductivity and oospore production by *Pythium aphanidermatum*

The effect of *Aspergillus terreus* isolate culture filtrate (CF) on electrolyte leakage of *Pythium aphanidermatum* was investigated using a conductivity meter (Manhas & Kaur, 2016; Halo, Al-Yahyai & Al-Sadi, 2018). Six conical flasks containing 200 ml of potato dextrose broth (PDB) were incubated with a four mm disk of *Aspergillus terreus* isolate mycelia at 28 °C for 14 days in an incubator shaker. At the end of the incubation period, *Aspergillus terreus* mycelia were separated from the CF using sterile filter paper followed by centrifugation at 10,000×*g*. Moreover, the CF was filtered using Ministar filter with 0.2 µm pore size. Further work was conducted as explained by Halo, Al-Yahyai & Al-Sadi (2018). The experiment was repeated twice.

The antifungal activity of *Aspergillus terreus* isolate was tested on oospore production. Petri dishes containing V8 agar (0.2 l of purified vegetable juice and 10 g of agar in 0.8 l of distilled water) were used as a control and V8 agar was mixed with *Aspergillus terreus* isolate CF at a concentration of 20%. After that, a three mm disk of *Pythium aphanidermatum* was placed onto Petri dishes and incubated at 28 °C for 3 weeks. Oospore number was calculated in 30 consecutive squares of a microscope at 40× magnification. The experiment was conducted using three replicates for the treatments and the control, according to Halo, Al-Yahyai & Al-Sadi (2018).

### Analysis of cellulase production by *Aspergillus terreus*

Measurement of total cellulase activity was done using the filter paper assay. The *Aspergillus terreus* isolate was grown in Basel medium of Mandels (Oberoi et al., 2010). The medium was incubated on a rotary shaker at 200 rpm and 28 °C for 10 days. 0.5 ml of fungal the CF was placed in test tubes containing one ml of sodium acetate buffer (50 mM, pH 4.8). Further assays were according to Halo, Al-Yahyai & Al-Sadi (2018). The absorbance was measured by a spectrophotometer at 540 nm (Ghose, 1987). Comparing the absorbance at 540 with glucose standard (0.2–5.2) mg/ml was done to calculate the reducing sugar produced in 1 h.

### Effect of *Aspergillus terreus* isolate on damping-off suppression and on growth of cucumber

The effect of *Aspergillus terreus* on Pythium damping-off disease of cucumber was studied using the following protocol. Three different treatments and a control were prepared. Each treatment and the control consisted of five pots (12-cm diameter) and six cucumber seeds (Jabber, F1) in each pot. Potting mix was added to each pot. A nine cm disc of *Pythium aphanidermatum* was placed on the surface of the potting mix of treatments 1 and 2, followed by covering it with two cm potting mix. A total of 25 ml of *Aspergillus terreus*, grown in 2.5% PDB for 10 days on a rotary shaker at 28 °C, was added to the second treatment. The third treatment was irrigated with *Aspergillus terreus* liquid culture (without *Pythium aphanidermatum*). Control pots received neither *Pythium aphanidermatum*, nor *Aspergillus terreus* treatments. Pots were incubated in a growth chamber for ten days under 80% relative humidity at 28 °C. The surviving seedlings of cucumber, seedling height, and fresh and dry weights were determined for each treatment/control. The experiment was repeated two times. Comparison of means was done using Tukey’s Studentized range test (SAS software, v.8).

## Results

### Antagonistic activity of fungal isolates against *Pythium aphanidermatum*

**Figure 1:**
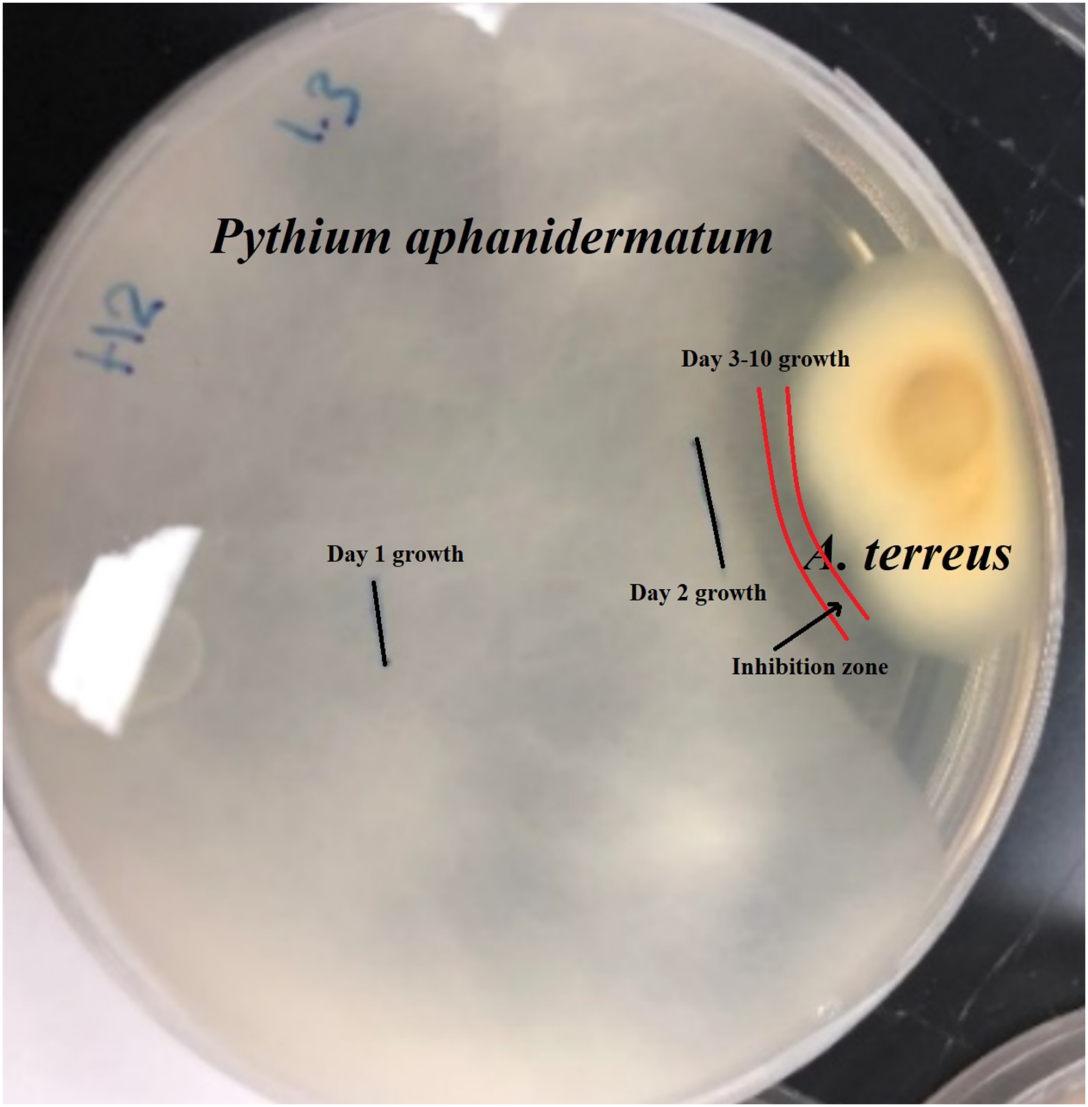
Inhibition zone produced between *Aspergillus terreus* and *Pythium aphanidermatum*.

Isolate H2 (*Aspergillus terreus*) produced an inhibition zone of 2.3 mm (Fig. 1). No further growth of *Pythium aphanidermatum* was detected, even after a month.

### Identification of *Aspergillus terreus* isolate

**Figure 2:**
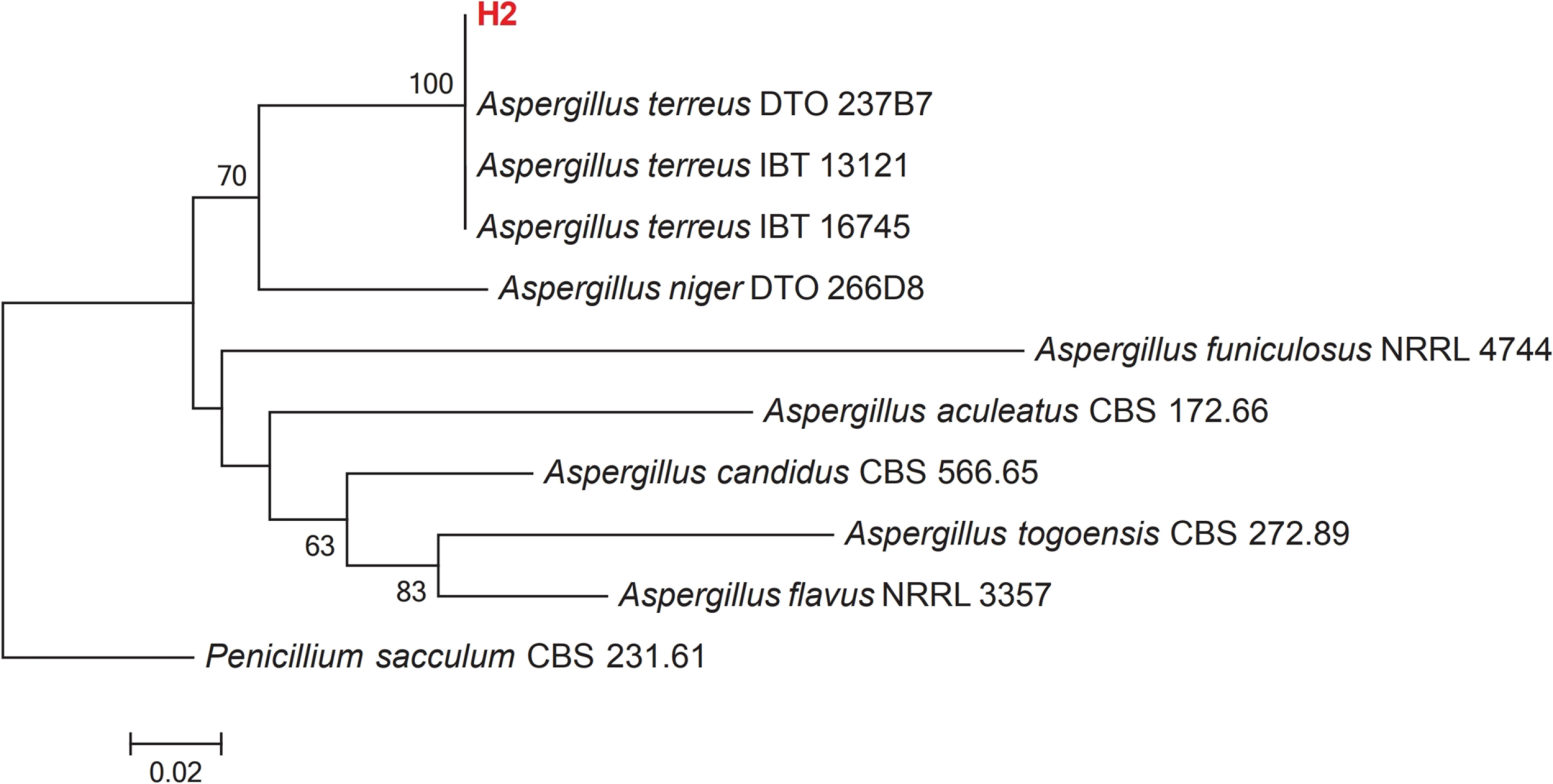
Maximum-likelihood phylogenetic tree inferred from ITS sequences of 11 taxa. Isolates of the present study are depicted in red bold lettering and bootstrap values above 50% are shown in the nodes. The tree is rooted to *Penicillium sacculum* (CBS 231.61).

Growth of culture of H2 isolate in PDA revealed that it belongs to the *Aspergillus* genus. Colonies of *Aspergillus* on PDA attained a diameter of up to 45 mm after 7 days at 25 °C. Colony texture is mostly fluffy and colonies are cinnamon brown and reverse cream-colored to light brown. Conidiophores are long, columnar hyaline and smooth; vesicles nearly globose, biseriate; phialides flask-shaped; conidia 1.5–2.5 µm in diameter. The obtained ITS rRNA sequence of the isolate was compared with 10 reference isolates from NCBI. The manually adjusted dataset comprised 506 characters, including gaps. The *Aspergillus* isolate recovered here (H2), clusters with other *Aspergillus terreus* isolates from GenBank with high bootstrap support (100%) (Fig. 2). Morphological and molecular analyses confirm that our isolate is *Aspergillus terreus*.

### Effect of *Aspergillus terreus* isolate on *Pythium aphanidermatum* hyphal morphology

**Figure 3:**
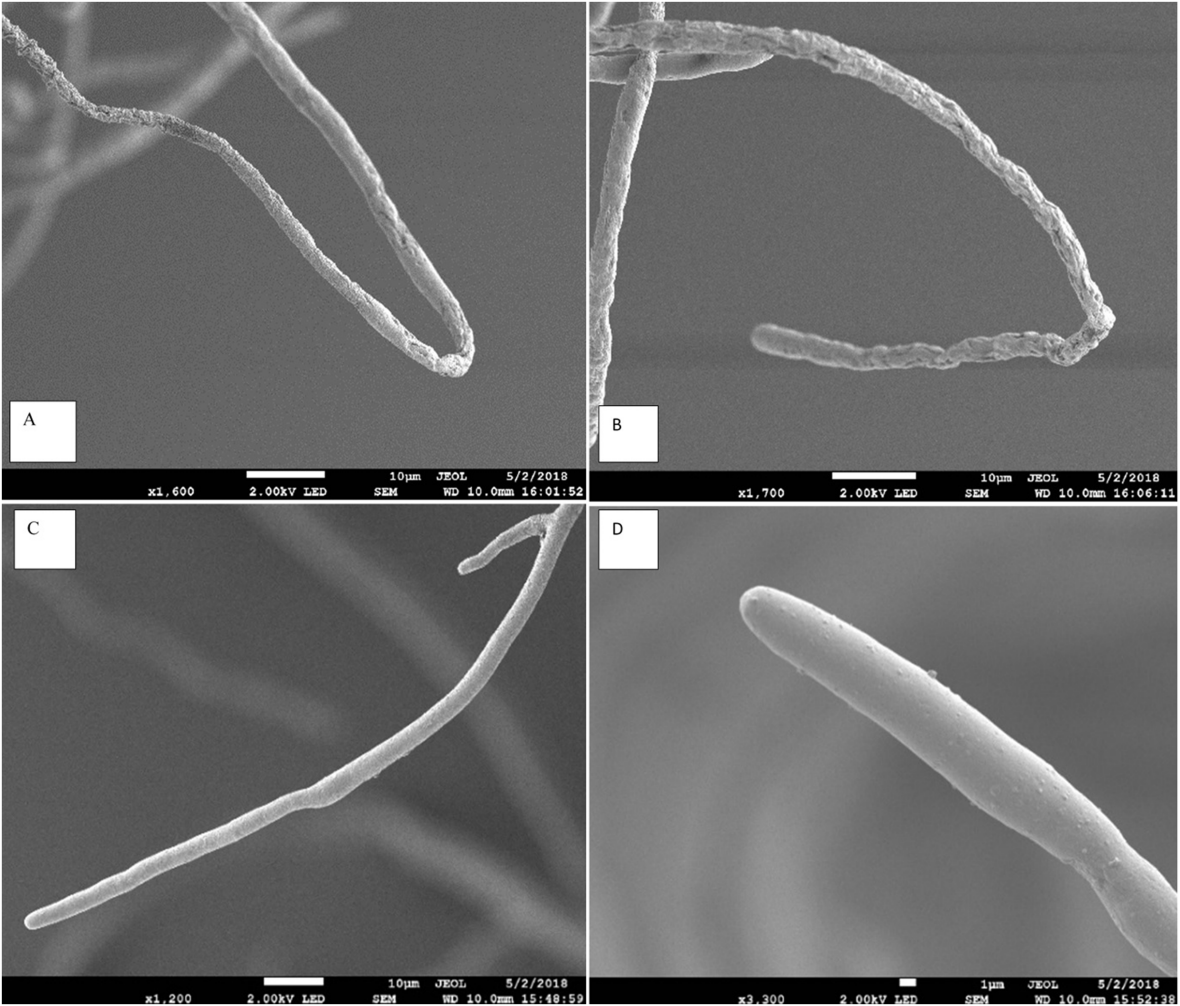
Effect of *Aspergillus terreus* on morphology of *Pythium aphanidermatum* hyphae using scanning electron microscope (SEM). (A & B) abnormal *Pythium aphanidermatum* hypha: shrunken and wrinkled under effect of *Aspergillus terreus*. (C & D) Normal hypha of Pythium aphanidermatum (control).

The scanning electron microscope clearly showed that the *Aspergillus terreus* isolate caused significant change in hyphal morphology of *Pythium aphanidermatum* compared with hypha of the control plate. Hypha from the inhibition zone area was shrunk, with deformations and groves, and it showed backward bendings (Fig. 3). The shrinking of hyphae indicated loss of material from the hypha. Bending in hyphae was at the hyphal tips, which was observed on hyphae facing the inhibition zone.

### Effect of *Aspergillus terreus* isolate on electrolyte leakage and production of oospores

Applying the CF of *Aspergillus terreus* on *Pythium aphanidermatum* resulted in significantly increased extracellular conductivity in comparison with the control after 24 h, with 0.22250 mS/cm for the CF of *Aspergillus terreus* and 0.06 mS/cm for control (*P* < 0.05). This result suggests that treating *Pythium aphanidermatum* mycelium with the CF of *Aspergillus terreus* results in electrolyte leakage. The production of oospores by *Pythium aphanidermatum* in CF of *Aspergillus terreus* (47 oospores) was significantly reduced in comparison with the control (161 oospores, *P* < 0.05).

### Analysis of cellulase activity by the *Aspergillus terreus* isolate

The standard graph was plotted with concentration of glucose on the *x*-axis and absorbance value at 540 nm on *y*-axis. Linear equation of glucose stander curve is *y* = 0.0297*x* + 0.2333, *R^2^* = 0.9542. The cellulase enzyme activity on filter paper was found to be 2.25 FPU/ml in the CF of *Aspergillus terreus* after 60 min of incubation. One filter paper unit (FPU) is defined as the amount of cellulase enzyme that liberates one µmol glucose per ml per minute.

### Effect of *Aspergillus terreus* on damping-off suppression and on growth of cucumber

**Figure 4:**
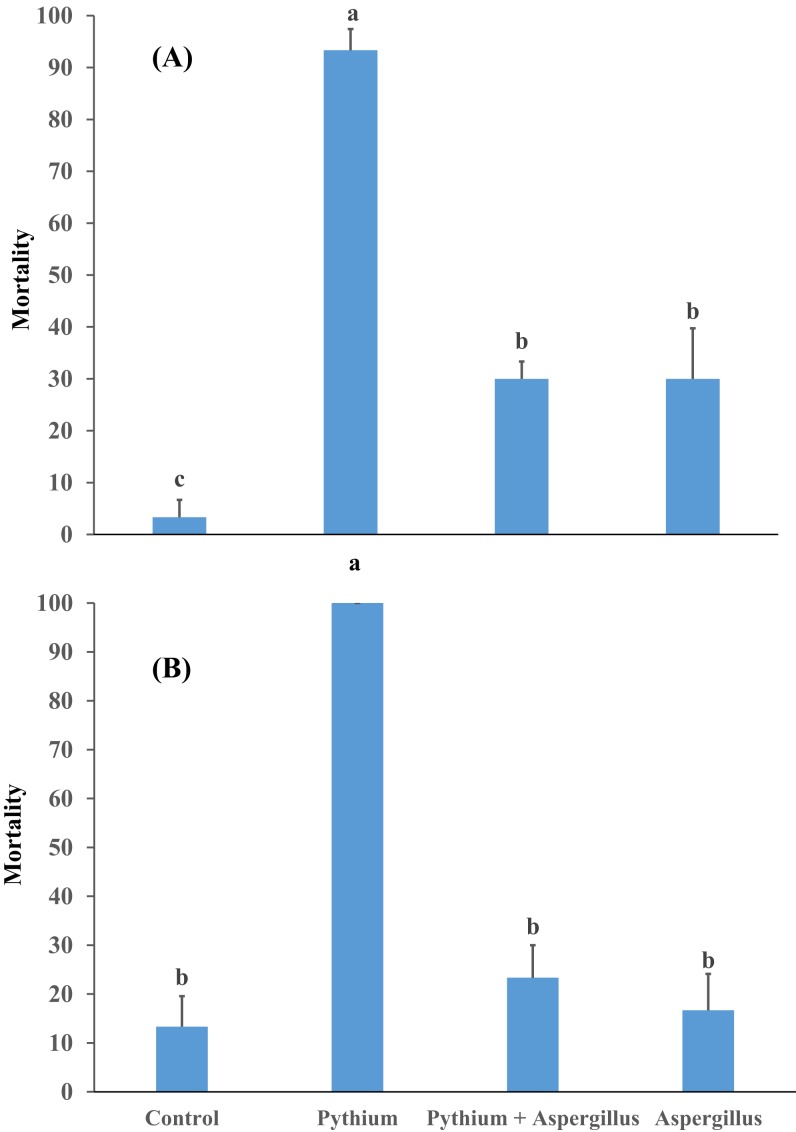
Effect of *A. terreus* on percent seedling mortality in cucumber inoculated with *P. aphanidermatum* ((A) trial 1, (B) trial 2). Columns with different letters indicate that they are significantly different from each other at *P* < 0.05 (Tukey’s Studentized range test, SAS v.8). Error bars are displayed for the different treatments.

**Figure 5:**
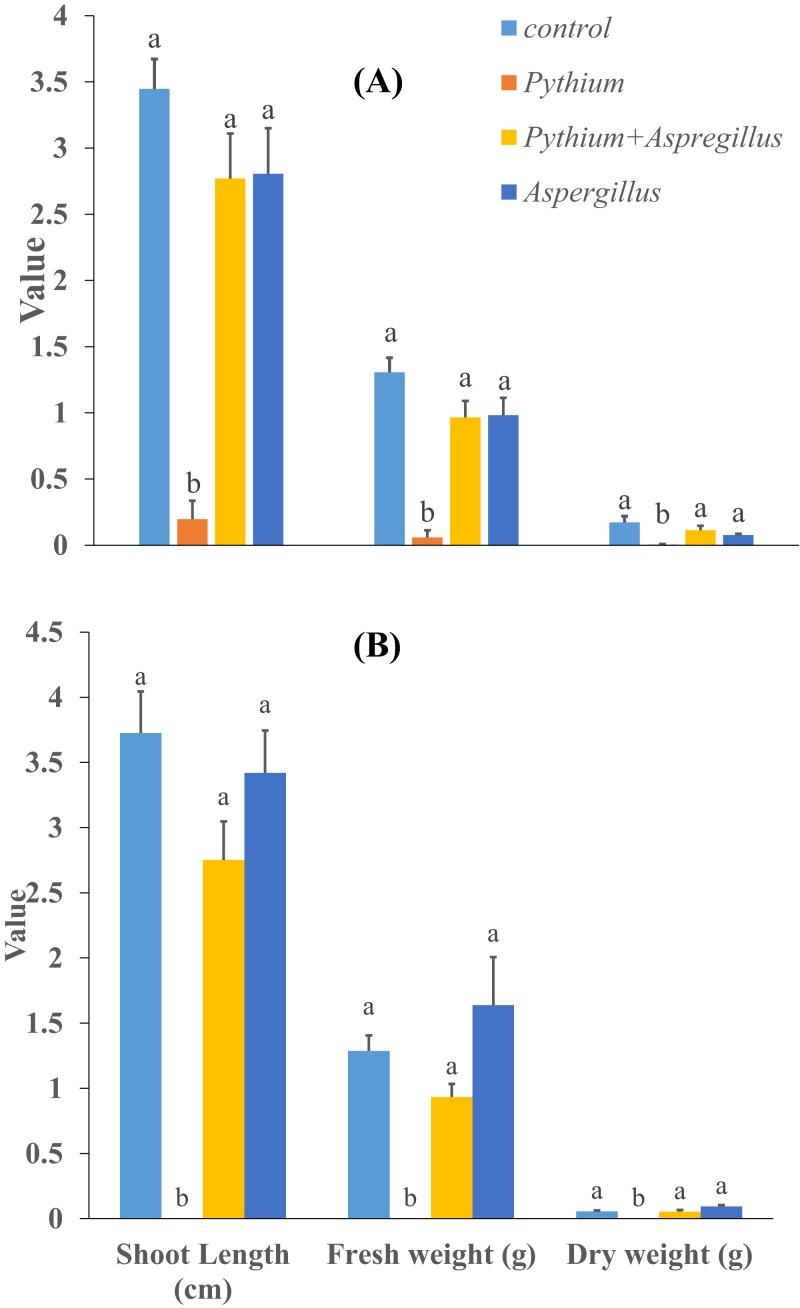
Effect of *Aspergillus terreus* on shoot length, fresh and dry weight of cucumber seedlings ((A) trial 1, (B) trial 2). Bars with different letters are significantly different from each other (Tukey’s Studentized range test, SAS). Error bars are displayed for the different treatments.

When cucumber seedlings inoculated with *Pythium aphanidermatum* were treated with *Aspergillus terreus*, the survival rate significantly increased by 70%, to levels similar to when *Aspergillus terreus* was used alone (Fig. 4). In addition, no significant effect was found of *Aspergillus terreus* on shoot length or dry and fresh weight of cucumber seedlings (Fig. 5).

## Discussion

The isolation of an antagonistic fungal isolate to *Pythium aphanidermatum* from *Avicennia marina* indicates that mangroves can serve as a source of microbes with potential biological applications. Previous studies have shown that fungal isolates from mangroves showed potent bioactivities. *Aspergillus* sp. D1 isolated from mangrove proved to produce L-asparaginase, which inhibits the formation of acrylamide compounds (Prihanto, Caisariyo & Pradarameswari, 2019). *Alternaria longipes* extract from mangrove exhibited antidiabetic activity for type 2 diabetes (Ranganathan & Mahalingam, 2019). Liu et al. (2017b) reported that some extracts of endophytic fungi from mangrove can inhibit lung cancer cell growth.

There is a recent move toward the search for antagonistic fungal isolates from mangrove against soil-borne fungal pathogens of crops. A study by Hamzah et al. (2018) revealed that some fungal isolates obtained from mangrove can inhibit the growth of the soil-borne fungus *Fusarium solani*. *Cladosporium oxysporum* isolated from mangrove was found to have biocontrol potential against *Rhizoctonia solani*, the cause of cucumber damping-off (Sureshkumar & Kavitha, 2019).

Our study showed that our *Aspergillus* isolate inhibited the growth of *Pythium aphanidermatum*. Identification of the isolate showed that it is *Aspergillus terreus*. *Aspergillus terreus* also resulted in changes in *Pythium aphanidermatum* hyphal morphology, where the affected hypha were shrunk, twisted, and with groves. This gave indication that the isolate not only inhibits growth but also causes abnormalities in the pathogen hypha. A previous study showed that *Aspergillus terreus* isolated from the rhizosphere of cucumber showed antagonistic activities against *Fusarium, Rhizoctonia*, and *Sclerotinia*-induced root diseases in cucumber (Lyu et al., 2015). Another study showed that some antagonistic fungal isolates inhibiting the growth of *Pythium aphanidermatum* can also result in morphological abnormalities in their hypha (Halo, Al-Yahyai & Al-Sadi, 2018).

The CF of the *Aspergillus terreus* isolate affected oospore production and resulted in electrolyte leakage from *Pythium* hyphae. This indicates the *Aspergillus terreus* utilizes several strategies to affect the growth and development of *Pythium aphanidermatum*. *Aspergillus terreus* was found to produce cellulase enzyme. Electrolyte leakage from *Pythium* hypha as well as deformations in the *Pythium* hypha might be related in part to the activity of the cellulase enzyme of *Aspergillus terreus*. Several studies indicated the effects of cellulase enzyme of the biocontrol agent, including *Aspergillus* spp., on pathogen hyphae (Madi et al., 1997; Ayyadurai et al., 2006; Gao et al., 2008; Lee, Tan & Ting, 2014; Sae-Lee & Boonmee, 2014; Halo, Al-Yahyai & Al-Sadi, 2018).

The use of *Aspergillus terreus* in *Pythium aphanidermatum*-infested soil significantly reduced damping-off disease of cucumber. This may be related to the effects of *Aspergillus terreus* on *Pythium aphanidermatum* growth, oospore production, and hypha development. Additionally, no negative effects were detected for *Aspergillus terreus* on cucumber growth. Similarly, previous studies showed that antagonistic microorganisms, including *Aspergillus terreus*, either have positive or no negative effects on the growth of cucumber and other crops (Elgorban, Bahkali & Al-Sum, 2013). However, in one trial, the survival rate of cucumber seedlings was higher in the control compared to soil infested with *Aspergillus terreus*. Although no disease was caused by *Aspergillus terreus* in cucumber, and since it was not consistent in the other trial, it may be related to some experimental factors other than pathogenicity of *Aspergillus terreus* on cucumber.

## Conclusions

Marine fungi received less attention than their terrestrial counterparts. Most of the previous work focused on fungi from soils or plants, with little attention been given to fungi from the marine environment (Al-Balushi et al., 2017; Chen et al., 2017; Dertli & Çon, 2017; Liu et al., 2017a; Yao et al., 2017; Huang et al., 2018; Perera et al., 2018; Yang et al., 2018). Although a previous study showed that *Aspergillus terreus* from desert plants can have antagonistic activities against *Pythium aphanidermatum* (Halo, Al-Yahyai & Al-Sadi, 2018), this study is the first to characterize a fungal isolate from mangrove (*Avicennia marina*) with antagonistic activity against *Pythium aphanidermatum*-induced damping-off of cucumber. This suggests that fungal antagonism is not affected by the origin of isolates, that is, terrestrial isolates and marine isolates of the same species can both show antagonism against terrestrial pathogens. Isolation of a fungal strain from mangrove with potent activity against a crop pathogen necessitate more future studies focusing on the potential activities of mangrove-associated fungal species. In addition, these studies should be coupled with conservation programs of the biologically-rich mangrove “forests.” Also, the ability of this isolate to interfere with germination of *Pythium aphanidermatum* spores should be investigated. Several *Aspergillus* species are known to have negative effects on humans through the production of different types of mycotoxins. Therefore, future studies should investigate the potential presence of negative effects of this isolate on humans or human food.

## Supplemental Information

10.7717/peerj.7884/supp-1 Supplemental Information 1 Raw data.

## Additional Information and Declarations

### Data Availability

The following information was supplied regarding data availability: The sequence data of the fungal isolate is available at GenBank: MK680140.
